# Outstanding Charge Mobility by Band Transport in Two-Dimensional Semiconducting Covalent Organic Frameworks

**DOI:** 10.1021/jacs.2c02408

**Published:** 2022-04-14

**Authors:** Shuai Fu, Enquan Jin, Hiroki Hanayama, Wenhao Zheng, Heng Zhang, Lucia Di Virgilio, Matthew A. Addicoat, Markus Mezger, Akimitsu Narita, Mischa Bonn, Klaus Müllen, Hai I. Wang

**Affiliations:** † Max Planck Institute for Polymer Research, Ackermannweg 10, Mainz D-55128, Germany; ‡ School of Science and Technology, Nottingham Trent University, Clifton Lane, Nottingham NG11 8NS, U.K.; § Organic and Carbon Nanomaterials Unit, Okinawa Institute of Science and Technology Graduate University, Okinawa 904-0495, Japan; ∥ Institute of Physical Chemistry, Johannes Gutenberg-University, Duesbergweg 10-14, Mainz 55128, Germany; ⊥ State Key Laboratory of Inorganic Synthesis and Preparative Chemistry, College of Chemistry and International Center of Future Science, Jilin University, Changchun 130012, P.R. China

## Abstract

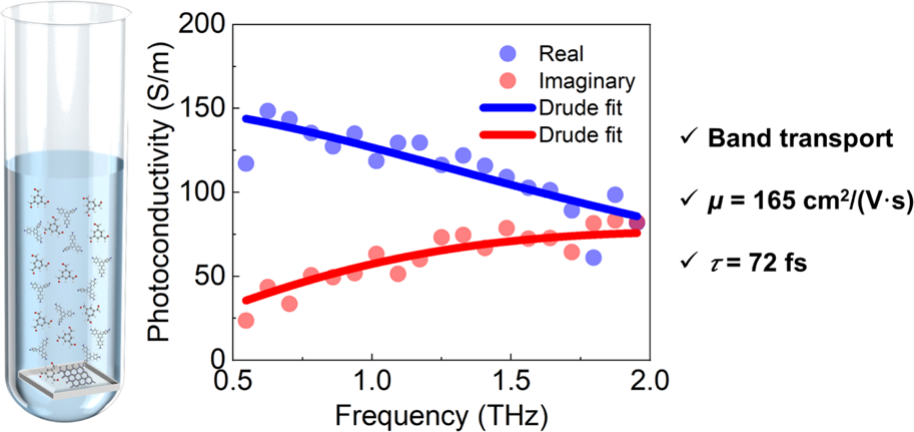

Two-dimensional covalent organic frameworks (2D COFs) represent a family of crystalline porous polymers with a long-range order and well-defined open nanochannels that hold great promise for electronics, catalysis, sensing, and energy storage. To date, the development of highly conductive 2D COFs has remained challenging due to the finite π-conjugation along the 2D lattice and charge localization at grain boundaries. Furthermore, the charge transport mechanism within the crystalline framework remains elusive. Here, time- and frequency-resolved terahertz spectroscopy reveals intrinsically Drude-type band transport of charge carriers in semiconducting 2D COF thin films condensed by 1,3,5-tris(4-aminophenyl)benzene (TPB) and 1,3,5-triformylbenzene (TFB). The TPB–TFB COF thin films demonstrate high photoconductivity with a long charge scattering time exceeding 70 fs at room temperature which resembles crystalline inorganic materials. This corresponds to a record charge carrier mobility of 165 ± 10 cm^2^ V^–1^ s^–1^, vastly outperforming that of the state-of-the-art conductive COFs. These results reveal TPB–TFB COF thin films as promising candidates for organic electronics and catalysis and provide insights into the rational design of highly crystalline porous materials for efficient and long-range charge transport.

## Introduction

Conventional amorphous semiconducting polymers exhibit localized charge transport properties with reported state-of-the-art charge carrier mobility on the order of 1 cm^2^ V^–1^ s^–1^, partially owing to local structural deformations originating from the disordered nature of polymer blends.^1^ Therefore, it is highly desirable to explore organic structures with a long-range order, whose extended electronic states are delocalized to facilitate band-like transport toward highly mobile organic electronics. In this regard, recently developed crystalline molecular semiconductors^2−4^ exhibit charge mobilities exceeding 10 cm^2^ V^–1^ s^–1^.

Two-dimensional covalent organic frameworks (2D COFs) with periodic nanochannels, abundant active sites, high chemical and thermal stability, and tailorable topologies have emerged recently as promising alternative electronic materials for solid electrolytes^5−10^ and optoelectronics.^11−16^ As salient characteristics, 2D COFs possess 2D polygon sheets along the *x*–*y* plane mimicking the graphene lattice and layer-stacked architectures along the *z*-direction resembling layered van der Waals materials. Although their long-range-ordered frameworks hold great promise for electronics,^17−23^ 2D COFs with high charge carrier mobility comparable to that of inorganic semiconductors remain scarce, and the underlying charge transport mechanism has remained largely elusive.

Since the advent of 2D COFs,^20^ many studies have been committed to tuning their charge transport properties by exploiting various subunits,^22,24^ linkages,^25−27^ and topologies.^28^ One representative study by Yaghi and co-workers^29^ demonstrated impressive charge mobilities of 8.0 and 3.1 cm^2^ V^–1^ s^–1^ in boronate ester and imine-linked porphyrin-based 2D COFs by integrating flash photolysis time-resolved microwave conductivity measurements (FP-TRMC) with time-of-flight (TOF) transients at different bias voltages. Follow-up works developed high-mobility 2D COFs by employing macromolecular building blocks,^30^ exploring π-conjugated linkages,^26,31^ regulating orientations,^32,33^ and doping.^34,35^ For instance, Dalapati et al*.*^28^ integrated a hexabenzocoronene (HBC) building block into the backbone of an imine-linked triangular COF, achieving a charge mobility of 0.7 cm^2^ V^–1^ s^–1^. Moreover, utilizing π-conjugated linkages, Guo et al*.*^26^ reported a charge mobility up to 4.2 cm^2^ V^–1^ s^–1^ in a phenazine-linked 2D COF using FP-TRMC combined with TOF transients at different bias voltages. Recently, Wang et al.^31^ have disclosed a record device-relevant mobility of ∼5 cm^2^ V^–1^ s^–1^ in field-effect transistors based on π-conjugated pyrazine-linked 2D COFs by the condensation of zinc-phalocyanine and *tert*-butylpyrene-tetraone. The mobility was further improved up to 22 cm^2^ V^–1^ s^–1^ by molecular iodine doping.^34^ Despite the impressive progress in developing high-mobility 2D COFs, the reported mobilities are far lower than those of their metal-based 2D counterparts, that is, 2D metal–organic frameworks (2D MOFs) with a record mobility over 200 cm^2^ V^–1^ s^–1^, as reported by Dong et al*.*^36^ Furthermore, according to recent spectroscopic studies,^31,34,37^ photogenerated charge carriers in 2D COFs are subject to the strong spatial confinement, likely originating from their small grain sizes. This is in stark contrast to the fully delocalized, band-like charge transport reported in the π–*d* conjugated porous Fe_3_(THT)_2_(NH_4_)_3_ MOFs.^36^ Finally, photogenerated charge carriers in all the 2D COFs reported till now^31,34,37^ exhibit short lifetimes on the order of sub-10 ps, likely due to charge trapping at defects. The charge localization and short carrier lifetime impede 2D COFs toward advanced electronics and optoelectronics, which calls for fundamental understanding of their intrinsic charge transport and recombination mechanisms.

Here, we report the first observation of band-like transport in highly crystalline 2D TPB–TFB COF thin films. Terahertz (THz) spectroscopy reveals the Drude response of photogenerated charge carriers, typical of band transport, with a charge scattering time of up to 70 fs. Our studies unveil an intuitive but critical parameter to ensure long-range, delocalized charge transport: crystallinity. Remarkably, our results disclose an exceptionally high charge mobility of 165 ± 10 cm^2^ V^–1^ s^–1^ in highly crystalline TPB–TFB COF thin films at room temperature, comparable to mobilities in inorganic semiconductors. Along with the band-like transport nature, the observed high mobility also originates from the largely reduced scattering events as demonstrated by the long charge scattering time of up to 70 fs. This value is superior to that of the previously reported 2D COFs, whose scattering times range from 30 to 50 fs (with non-Drude transport characteristics).^31,34,37−39^ Temperature-dependent THz photoconductivity measurements confirm the band-like charge transport in TPB–TFB COF thin films, by showing a negative temperature dependence of the mobility. Furthermore, there remains considerable room for further enhancing the mobility, as impurity scattering plays a nontrivial role in limiting the charge mobility. Our results are of importance for 2D COFs toward electronics, as they not only unveil the intrinsic charge transport mechanism in 2D COFs but also demonstrate the possibility of producing 2D COFs with high mobilities and the relevance of highly crystalline TPB–TFB COF thin films for organic electronics.

## Results and Discussion

**Figure 1:**
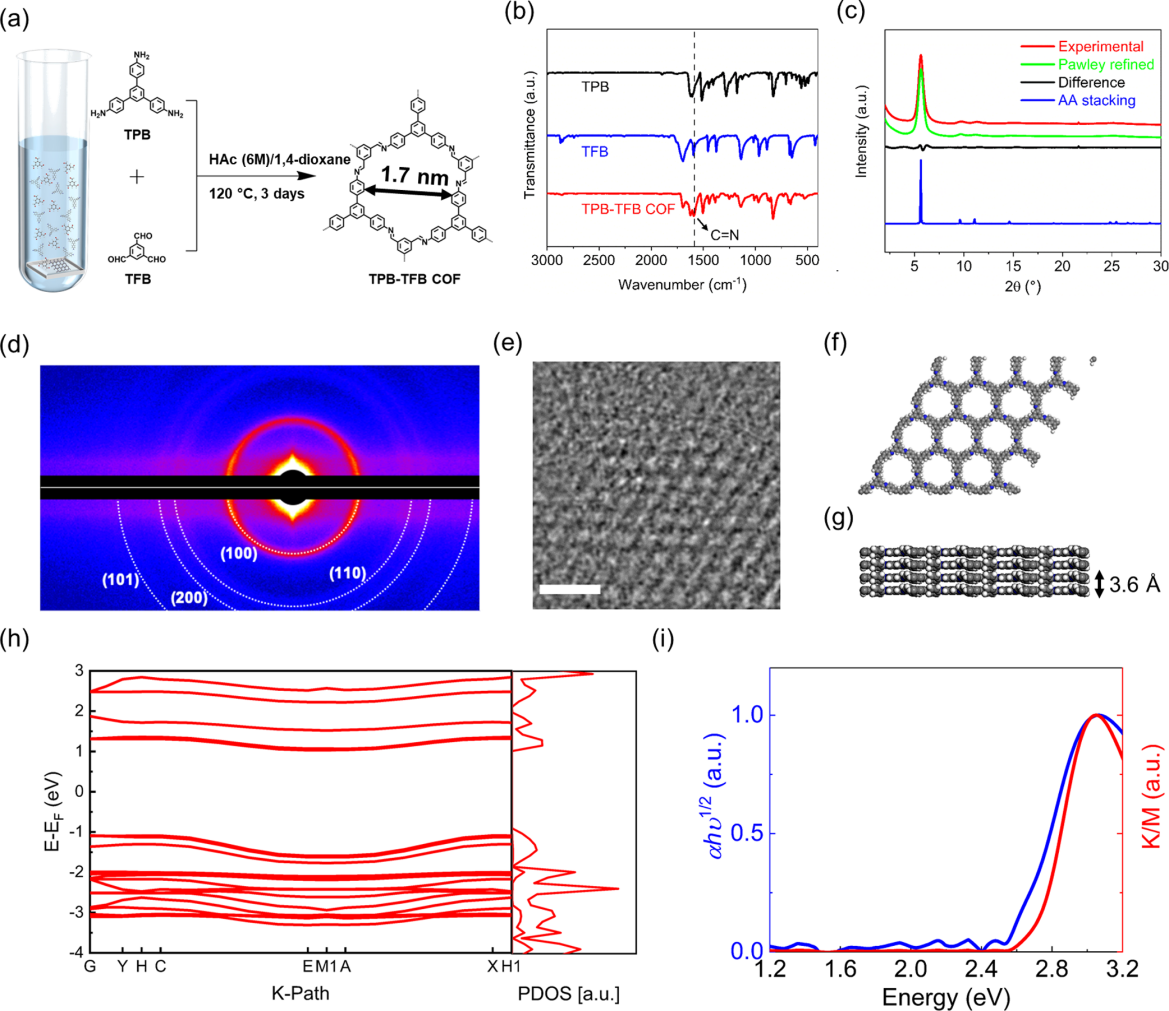
Synthesis and characterization of TPB–TFB COFs. (a) Schematic of the synthesis of TPB–TFB COF powder and thin films. (b) FTIR spectra of TPB (black), TFB (blue), and TPB–TFB COF (red). (c) PXRD patterns of TPB–TFB COF powder: experimental pattern (red), Pawley refined pattern (green), differences between the experimental and Pawley refined patterns (black), and the simulated pattern for the AA-stacking mode (blue). (d) 2D X-ray scattering pattern of a 3 μm thick TPB–TFB COF thin film recorded at a grazing incident angle of 0.2°. (e) TEM image of TPB–TFB COF showing hexagonal pore structures (scale bar: 2 nm). Reconstructed crystal structures of the (f) top and (g) side views of TPB-TFB COFs. The 2D layers are stacked along the out-of-plane direction with an interlayer spacing of 3.6 Å. (h) Calculated electronic structure of the AA-stacked TPB–TFB COF. The left and right panels display the calculated electronic band structure and the corresponding projected density of state (PDOS), respectively. (i) The blue line represents the Tauc plot of TPB-TFB COF thin film, and the red line stands for the Kubelka–Munk-transformed reflectance spectrum of the TPB–TFB COF powder.

We synthesize imine-linked 2D hexagonal TPB–TFB COFs via a facile and mild solvothermal polymerization approach^40,41^ driven by the condensation of *C*_3_-symmetric 1,3,5-*tris*(4-aminophenyl)benzene (TPB) and *C*_3_-symmetric 1,3,5-triformylbenzene (TFB) monomers. As schematically depicted in Figure 1a, the polymerization of TPB and TFB monomers is performed in a mixture of dioxane (1.0 mL) and acetic acid solution (HAc, 6 M, 0.1 mL) at 120 °C for 3 days. Fused silica substrates are put inside the solution to allow the formation of TPB–TFB COFs in two distinct forms: powder in the solution and thin films on the fused silica substrates (see details in SectionS1). The Fourier-transform infrared (FTIR) spectrum (Figure 1b) supports the formation of TPB–TFB COFs by the emergence of imine stretching vibration at 1630 cm^–1^. Powder X-ray diffraction (PXRD) pattern of the TPB–TFB COF powder (red line, Figure 1c) shows characteristic diffraction peaks at 2*θ* = 5.6, 9.8, 11.3, and 25.2°, which are assigned to the (100), (110), (200), and (001) facets, respectively. The strong reflection at 2θ = 5.6° indicates a structural periodicity of *d* = 2*π*/*q* ∼ 1.6 nm (see Figure 1a). Assuming a hexagonal symmetry, this corresponds to a lattice constant of *a* = 2/3*d* ∼ 1.8 nm. Indeed, the Pawley refined PXRD pattern (green line, Figure 1c and TableS1) using the *P1* space group and unit cell parameters of *a* = 18.8 Å, *b* = 18.7 Å, *c* = 3.61 Å, α = β = 90°, and γ = 120° (residuals *R*_wp_ = 4.89%, *R*_p_ = 3.69%) well reproduces the experimentally observed PXRD profile, as evidenced by their negligible difference (black line, Figure 1c). The diffraction peak at 2θ = 25.2° originating from the (001) facet suggests an ordered stacking along the out-of-plane direction with an interlayer spacing of 3.6 Å. Employing the density-functional tight-binding (DFTB+) method with the mio-1-1 parameter set, we find that the experimental PXRD profile matches well with the simulated AA-stacking mode (blue line, Figure 1c and Table S2) rather than the simulated AB-stacking one (FigureS1 and Table S3) in terms of the peak positions and intensities. The 2D X-ray scattering pattern of the thin film (Figure 1d) not only reproduces the pronounced (100), (110), and (200) Bragg reflections of the powder but also shows a weak signal around 2θ = 25.0° for the (101) Bragg reflection that is characteristic of the AA-stacking mode. Furthermore, the peak width for the (100) Bragg reflection of the 3 μm thick film (Δ*q*/*q* ∼ 0.12) is smaller than that of the powder (Δ*q*/*q* ∼ 0.14). A further significant decrease in the innermost reflection peak widths is observed for a 200 nm thick thin film (Figure S2). According to the Scherrer equation, Δ*q*/*q* is inversely proportional to the crystallite size. These results, therefore, suggest that the crystallite size in thin films is larger than that in the powder.

The formation of TPB–TFB COF is further confirmed by high-resolution transmission electron microscopy (HR-TEM). Figure 1e shows a TEM image of the periodic hexagonal patterns for an exfoliated TPB–TFB COF thin film, where the obtained contrast matches well with an image for the simulated AA-stacking TPB–TFB COF^42^ (Figure S3a). Figure S3b visualizes a stacked-layer structure with an interlayer distance of 3.6 Å (Figure S3c), in good agreement with the PXRD data. Moreover, TEM images with periodic patterns at different intervals are obtained and reproduced well by the simulations generated from the AA-stacking TPB–TFB COF (Figure S3d–g). These results indicate a face-to-face arrangement of the discrete 2D COF sheets, forming periodically ordered and unidirectional hexagonal π-columns along the out-of-plane direction (Figure 1f,g). Nitrogen sorption isotherm measurements are taken at 77 K to evaluate the porosity of TPB–TFB COF powders and reveal a Brunauer–Emmett–Teller specific surface area of 1232 m^2^ g^–1^ (see Figure S4a). The average pore size is calculated as 1.7 nm using the nonlocal density functional theory (NLDFT) method (FigureS4b). Figure 1h displays the calculated electronic structure of TPB–TFB COF and the corresponding PDOS, demonstrating its semiconducting nature with an indirect band gap of ∼2.1 eV. Figure 1i compares the Tauc plot of the TPB–TFB thin film and the Kubelka–Munk-transformed reflectance spectrum of the TPB–TFB powder. Despite their different morphologies, both TPB–TFB thin film and powder exhibit an absorption onset of ∼2.6 eV and a maximum absorption at ∼3.0 eV. The hole mass (*m*_h_) and electron mass (*m*_e_) inferred from the energy band diagram are 2.04 *m*_0_ and 1.26 *m*_0_, respectively, giving rise to a reduced electron–hole mass of m* = 0.78 *m*_0_ (with *m*_0_ representing the electron rest mass).

**Figure 2:**
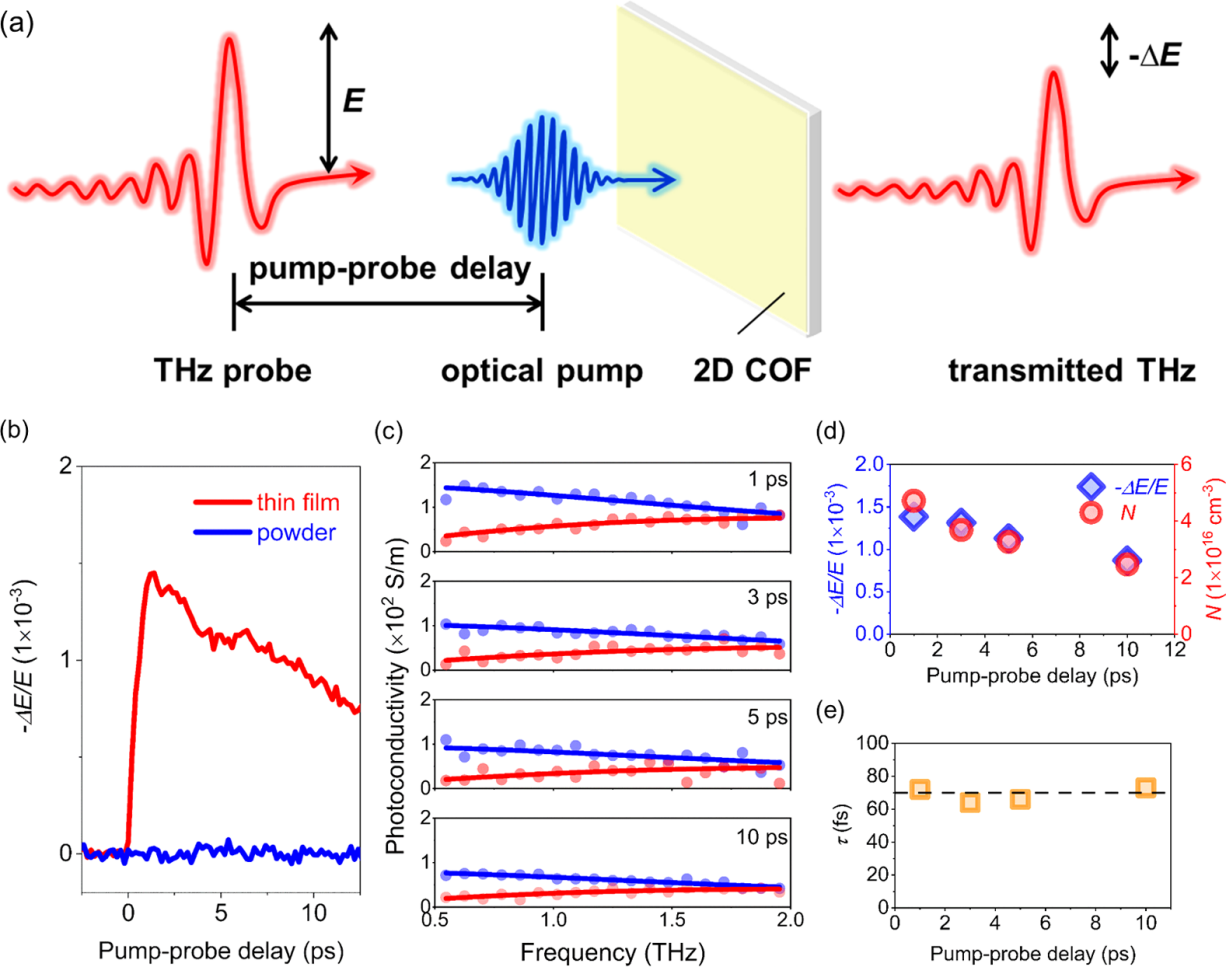
Ultrafast THz photoconductivity of TPB–TFB COF. (a) Schematic of the optical pump-THz probe spectroscopy. (b) Time-resolved THz photoconductivity of TPB–TFB COFs in the forms of a thin film (sample A) and powder. The samples are photoexcited by a femtosecond laser pulse (λ = 400 nm, absorbed pump fluence = 22 μJ/cm^2^), and their photoconductivity is probed by a single-cycle THz pulse with ∼1 THz bandwidth under dry N_2_ flow. (c) Frequency-resolved complex THz photoconductivity of the TPB–TFB COF thin film at different pump–probe delays following optical excitation. The blue and red dots represent the real and imaginary parts of the complex THz photoconductivity, respectively. The blue and red solid lines correspond to the Drude model describing the real and imaginary components of the complex THz photoconductivity, respectively. (d) Pump-induced changes in the transmitted THz electrical field (−Δ*E*/*E*, blue diamonds, left *y*-axis) and charge carrier densities (*N*, red circles, right *y*-axis) obtained from the Drude fits at different pump–probe delays. (e) Charge scattering times (τ) obtained from the Drude fits at different pump–probe delays. The black dashed line reflects the average of the four obtained charge scattering times (orange squares) at different pump–probe delays.

To elucidate the dynamics and transport properties of photogenerated charge carriers in semiconducting TPB–TFB COFs, we measure their time-resolved photoconductivity by optical pump–THz probe (OPTP) spectroscopy (see details in the Supporting Information). In a typical OPTP measurement, as schematically shown in Figure 2a, we optically inject charge carriers into TPB–TFB COFs by above band-gap excitations using ultrafast femtosecond laser pulses with a photon energy of 3.1 eV (λ = 400 nm). The pump-induced conductivity, that is, photoconductivity (Δσ) of charge carriers, is subsequently probed by a single-cycle, collinearly propagating THz pulse (with a peak electric field strength of *E*). The pump-induced, relative change in the transmitted THz electric field, −Δ*E*/*E*, is linearly proportional to Δσ.^43^ Following optical excitations, photogenerated charge carriers in the TPB–TFB COF thin film (red line, Figure 2b) exhibit large photoconductivity with a lifetime on the order of 10 ps. Note that Δσ is expressed as Δσ = *n*·*e*·μ, where *n*, *e*, and μ represent the photogenerated charge carrier density, elementary charge, and charge mobility, respectively. Our observation of large photoconductivity is remarkable, in particular taking into account the nearly transparent nature of the thin film (with an absorbance of ∼0.02 at *hν* = 3.1 eV) and thus a low density of charge carriers *n*. This directly provides the first indication of a large μ in the TPB–TFB COF thin film. As for the charge carrier lifetime, further extending the pump–probe delay window (see FigureS5) reveals that the photoconductivity decays in a biexponential fashion characterized by fast and slow relaxation components with time constants of *t*_1_ = 10 ± 1 ps and *t*_2_ = 131 ± 10 ps, respectively. The fast and slow relaxation components can be tentatively assigned to trapping and electron–hole recombination processes, respectively. The weighted fast and slow relaxation components yield an averaged charge carrier lifetime of *t* ∼ 40 ps, significantly longer than that of the other COFs characterized by the same technique,^31,34,37−39^ where the photoconductivity becomes negligible within sub-10 ps. To explore the role of crystallinity in determining the charge transport properties, we compare thin-film photoconductivity with that of the TPB–TFB COF powder (the sample morphology that has been extensively used in previous photoconductivity measurements). Under the same absorbed photon density, the TPB–TFB COF powder (Figure 2b, blue line) shows no photoconductivity within the noise level of the measurements, indicating that the charge mobility of the TPB–TFB powder is orders of magnitude lower than that of the film (by comparing the noise level of the data to the peak photoconductivity of the film sample). The dramatic photoconductivity difference between the thin film and the powder points out the critical role of crystallinity in determining the charge carrier lifetime and conductivity of 2D COFs.

To further confirm the high mobility of photogenerated charge carriers in TPB–TFB COF thin films and investigate their transport mechanism, we performed frequency-resolved complex photoconductivity [Δσ(ω)] measurements at varied pump–probe delays (i.e., 1, 3, 5, and 10 ps) using THz time-domain spectroscopy (THz-TDS, see details in the Supporting Information). As shown in Figure 2c, in all the cases, Δσ(ω) is in accordance with the spectral responses arising from delocalized free charge carriers that are typically observed in the crystalline inorganic materials^44−47^ and can be well described by the Drude model, following eq 1
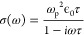
1where ω*_p_*, ε_0_, ω, and τ represent the plasma frequency, vacuum permittivity, angular frequency, and charge scattering time, respectively. Furthermore, the charge carrier density can be obtained from the inferred plasma frequency by eq 2
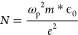
2where *N* and *e* stand for the charge carrier density and elementary charge, respectively.

The Drude fits to Δσ(ω) at different pump–probe delays depict the evolution of microscopic charge transport properties (i.e., *N* and τ). As shown in Figure 2d, the inferred *N* follows the same trend with −Δ*E*/*E* (and thus Δσ) during the photoconductivity decay, indicating that the decrease in *N* is responsible for the decay of Δσ, as consequences of the trapping and electron–hole recombination processes. Furthermore, the inferred τ is almost unchanged at the four pump–probe delays, as displayed in Figure 2e, giving rise to an averaged charge scattering time τ̅ = 72 fs (dashed line, Figure 2e). This suggests that τ is independent of *N* in the measured charge carrier density range (∼3–5 × 10^16^ cm^–3^). Using τ̅= 72 fs and m* = 0.78 *m*_0_ obtained from the DFT calculation, we infer the mobility of photogenerated charge carriers in TPB–TFB COF thin films to be 165 ± 10 cm^2^ V^–1^ s^–1^, following μ = *e*τ̅/m*. To the best of our knowledge, our results represent the first observation of the Drude transport of charge carriers in 2D COFs. Furthermore, the estimated mobility constitutes a record high in COFs and is almost one order of magnitude higher than the other reported conductive 2D COFs^31,34,37−39,48^ (see Table S5). Note that the obtained mobility combines the contributions of in- and out-of-plane conduction. Further efforts to control the layer orientation of 2D COFs, for example, by pre-organizing them on the water surface by exploiting the π–π interaction and hydrophobicity of monomers^49^ and choosing suitable substrates for COF growth,^41^ are required to disentangle the in- and out-of-plane conduction by THz spectroscopy. Such experiments are critical for providing insights into the potential anisotropic charge transport properties of 2D COFs. The intrinsically high charge mobility and the Drude-type charge transport observed here indicate that band transport prevails in the TPB–TFB COF thin films. The relatively long charge carrier lifetime and outstanding mobility facilitate the efficient and long-range transport of charge carriers in TPB–TFB COF thin films and lead to a diffusion length exceeding 100 nm (see details in SectionS9). The superior charge transport properties of TPB–TFB films over other molecular and polymeric materials (see Table S6) make them promising candidates for organic electrodes, electronics, and catalysis.

**Figure 3:**
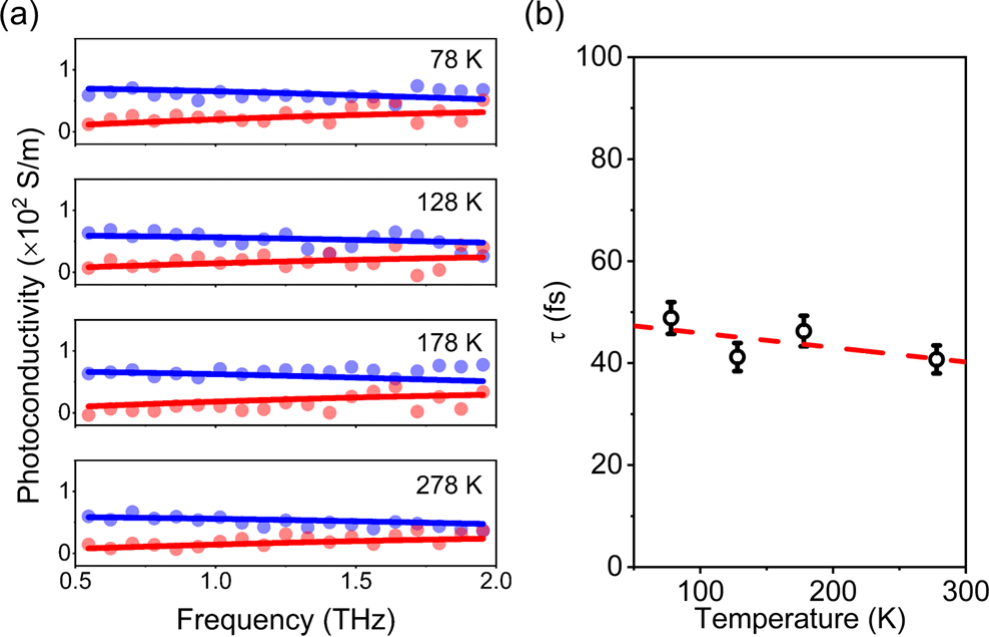
Temperature dependence of THz photoconductivity and scattering time of the TPB–TFB COF thin film. (a) Frequency-resolved complex THz photoconductivity of the TPB–TFB COF thin film (sample B) measured at 1 ps following optical excitation at different temperatures. The blue and red solid lines correspond to the Drude model describing the real and imaginary components of the complex THz photoconductivity, respectively. (b) Charge scattering times (*τ*) obtained from the Drude fits shown in (a). The red dashed line represents a linear fit to guide the eyes.

To provide further insights into the charge transport mechanism in the TPB–TFB COF thin films, we perform temperature-dependent frequency-resolved photoconductivity measurements spanning from 278 to 78 K. As shown in Figure 3a, the frequency-resolved photoconductivity retains the key signature of Drude-type transport at all the measured temperatures. Figure 3b shows the charge scattering time extracted from the Drude fits as a function of temperature (*T*). The charge scattering time exhibits a weak temperature dependence, increasing slightly with a decrease in *T*. Such dependence is qualitatively in line with band transport, as freezing out phonons at lower *T* reduces charge scattering. Impurity scattering likely weakens the temperature dependence of the mobility.^36,50,51^ The presence of defects is further evidenced by a correlation between the charge scattering time and charge carrier lifetime in our samples: compared to sample A used for the OPTP studies (shown in Figure 2), we noticed a reduced charge scattering time at room temperature (from 72 to 41 fs) in sample B used for the temperature-dependent measurements. This is accompanied by a reduction in the charge carrier lifetime (Figure S6). As such, despite being already high, the estimated mobility represents the lower bound that appears to be limited by defects and may be further improved by, for example, passivating defects by doping and screening of charged impurities through dielectric engineering.^34,52^ Furthermore, we expect that further enhancement of the charge transport properties can be achieved by increasing the conjugation within the layers and incorporating donor–acceptor structures into the backbones to enable the synthesis of 2D COFs promising for advanced optoelectronic applications.

## Conclusions

In summary, we report the first observation of Drude-type band transport of photogenerated charge carriers in 2D COFs and reveal the critical role of crystallinity in determining the charge transport properties. The highly crystalline TPB–TFB COF thin films exhibit a long scattering time beyond 70 fs at room temperature, corresponding to a record mobility of 165 ± 10 cm^2^ V^–1^ s^–1^ for 2D COFs. The outstanding mobility, together with the much-enhanced charge carrier lifetime compared to other 2D COFs (∼40 *vs* sub-10 ps), enables relatively efficient and long-range charge transport that holds great promise for organic optoelectronics, green catalysis, and chemiresistive sensing. Our results not only provide insights into the intrinsic charge transport mechanism in 2D COFs but also demonstrate the possibility of producing high-mobility 2D COFs through the rational design of their chemical structures and fine control of their crystallinity.

## Supporting Information Available

Experimental details and characterizations of the TPB-TFB COFs (PDF)
