# Supplementary material for: Outstanding Charge Mobility by Band Transport in Two-Dimensional Semiconducting Covalent Organic Frameworks
Source: J Am Chem Soc. 2022 Apr 14;144(16):7489–96. doi: 10.1021/jacs.2c02408 (PMC9052747; doi:10.1021/jacs.2c02408)
Supplement: Supplementary file 1 — ja2c02408_si_001.pdf [file ja2c02408_si_001.pdf]

Supporting Information

Outstanding Charge Mobility by Band Transport in Two-Dimensional Semiconducting Covalent Organic Frameworks

Shuai Fu[1]†, Enquan Jin[1,5]†, Hiroki Hanayama[3], Wenhao Zheng[1], Heng Zhang[1], Lucia Di Virgilio[1], Matthew A. Addicoat[2], Markus Mezger[1], Akimitsu Narita[1, 3], Mischa Bonn[1], Klaus Müllen[1,4]* and Hai I. Wang[1]**

[1]Max Planck Institute for Polymer Research, Ackermannweg 10, D-55128 Mainz, Germany

[2]School of Science and Technology, Nottingham Trent University, Clifton Lane, Nottingham NG11 8NS, UK

[3]Organic and Carbon Nanomaterials Unit, Okinawa Institute of Science and Technology Graduate University, Okinawa, 904-0495, Japan

[4]Institute of Physical Chemistry, Johannes Gutenberg-University, Duesbergweg 10-14, Mainz 55128, Germany

[5]State Key Laboratory of Inorganic Synthesis and Preparative Chemistry, College of Chemistry and International Center of Future Science, Jilin University, Changchun 130012, P.R. China

*Corresponding authors: bonn@mpip-mainz.mpg.de; muellen@mpip-mainz.mpg.de; wanghai@mpip-mainz.mpg.de

†These authors contributed equally to this work.

Table of contents:

Section S1. Experimental Procedures

Section S2. XRD pattern of the simulated AB-stacking TPB-TFB COF

Section S3. 2D X-ray scattering patterns of TPB-TFB COF thin films

Section S4. High-resolution transmission electron microscopic (HR-TEM) analysis of TPB-TFB COF

Section S5. Nitrogen sorption isotherms of TPB-TFB COF

Section S6. Optical-pump THz-probe (OPTP) spectroscopy

Section S7. Estimation of charge carrier lifetime

Section S8. Comparison of different TPB-TFB thin films

Section S9. Estimation of charge carrier diffusion length

Section S10. Comparison of charge mobilities of 2D COFs

Section S11. Comparison of charge transport properties of molecular and polymeric materials characterized by THz spectroscopy

Section S1. Experimental procedures

General Methods:

Unless otherwise noted, all reagents were purchased from commercial chemical companies and directly used without further purification. All reactions were carried out under vacuum with the standard Schlenk line technique. UV-vis absorption spectra were recorded on a Perkin-Elmer Lambda 900 spectrometer. Fourier-transform infrared spectra were measured on a Bruker TENSOR II FTIR spectrometer. Each sample was measured with a scan number of 64 and the background was subtracted. Powder X-ray diffraction (PXRD) patterns were recorded on a Rigaku SmartLab X-ray diffractometer by placing the powder samples on the glass substrates. The measurement was performed from $2\theta = 2.0°$ to 30° with an increment if 0.02°. XRD of the COF thin film was measured at a Rigaku SmartLab diffractometer (9kW rotating Cu anode) equipped with a HyPix-3000 2D photon counting X-ray detector at a sample-detector distance of 100 mm. The scattering pattern was recorded at a gracing incident angle of 0.2° with 16 h exposure. Nitrogen gas sorption curves were collected on a Micrometrics TriStar II Plus gas sorption instrument. Before measurement, powder samples were degassed in vacuum at 120 °C for 6 h. The Brunauer-Emmett-Teller (BET) approach was employed to evaluate the surface areas. Pore volume was calculated based on the nitrogen gas sorption curve by the non-local density functional theory (NLDFT) model. Molecular modeling and Pawley refinement were conducted using Reflex, a software package for crystal determination according to the XRD pattern, implemented in MS modeling version 4.4 (Accelrys Inc.). Pawley refinement was used to optimize the crystalline lattice parameters iteratively until the R_P and R_{WP} values converge. Geometries of monolayer, AA- and AB-stacking TPB-TFB-COF were calculated using Density Functional Tight Binding as implemented in DFTB+ version 20.1. All atom pairs were described using standard parameters from the mio-1-1 parameter set. Following the geometric optimization, the band structure, density of states (DOS), and effective mass were calculated using the 3rd order DFTB and the 3ob-3-1 parameter set. All the property calculations were undertaken in AMS-DFTB [AMS DFTB 2020, SCM, Theoretical Chemistry, Vrije Universiteit, Amsterdam, The Netherlands]. HR-TEM analysis was conducted on ThermoFisher Titan G2 electron microscope, operated at 200 kV. This microscope was equipped with a Schottky XFEG electron source, S-TWIN objective lens, image Cs-corrector (CEOS GmbH), with Gatan 2k×2k UltrascanXP1000 camera. A Fischione 2020 single tilt tomography holder was used for this work. TEM grids precoated with a lacy carbon film (NS-C15, pore size 1.5 – 8 μm) and with a continuous amorphous carbon film (UHR-C10) were purchased from Okenshoji Co., Ltd.. TEM

simulation images were generated by using a multi-slice prodedure implemented in the ELBis software.[1]

Synthetic Details:

Synthesis of TPB-TFB-COF powder. TPB was purified by recrystallization from ethanol for several times prior to use. A 15-mL microwave tube containing TPB (20 mg, 0.057 mmol), TFB (9.2 mg, 0.057 mmol), and 1,4-dioxane (1 mL) was sonicated for 30 s and degassed through three freeze-pump-thaw cycles before sealing under vacuum. Acetic acid (6 M, 0.1 mL) was then added. The tube was sealed and heated at 120 °C for 3 days. After cooling to room temperature, the resulting light-yellow precipitate was filtered and washed with tetrahydrofuran (THF) and acetone for several times. The powder was dried under vacuum at 60 °C for 12 h to produce TPB-TFB-COF in 92% yield.

Synthesis of TPB-TFB-COF thin film on the fused silica substrate. A 15-mL microwave tube containing TPB (25 mg, 0.057 mmol), TFB (9.2 mg, 0.057 mmol), and 1,4-dioxane (1 mL) was sonicated for 30 s. Afterwards, a fused silica substrate was added and the solution was degassed through three freeze-pump-thaw cycles before sealing under vacuum. Acetic acid (6 M, 0.1 mL) was then added. The tube was sealed and heated at 120 °C for 3 days. After cooling to room temperature, the fused silica substrate with light yellow thin film was submerged in anhydrous THF and acetone for three times. The thin film was dried under vacuum at room temperature for 12 h.

Section S2. XRD pattern of the simulated AB-stacking TPB-TFB COF

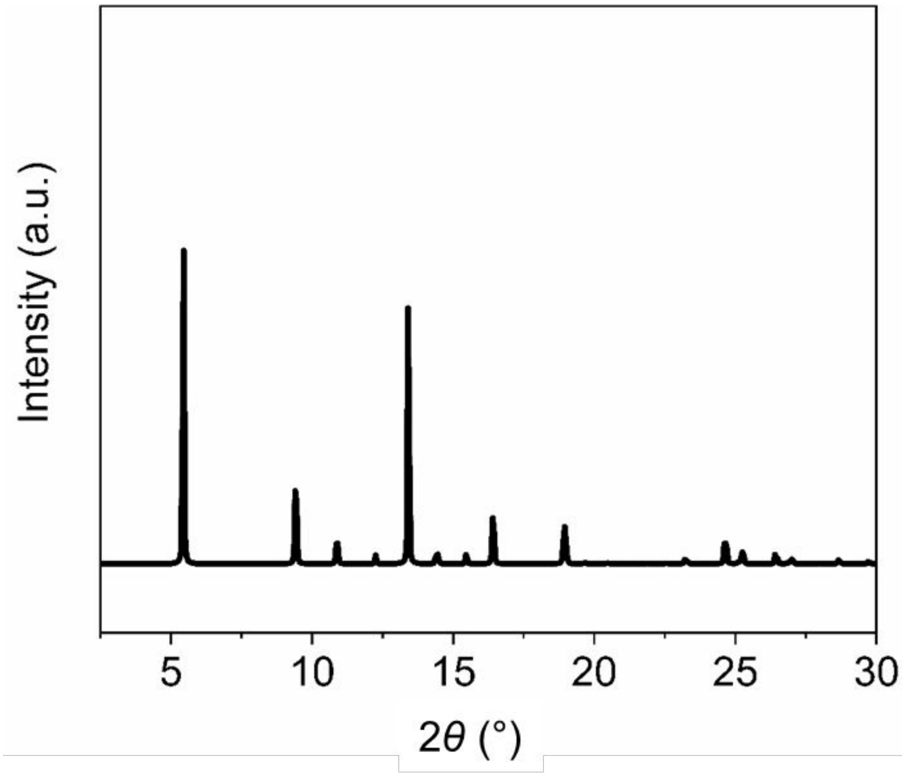

Figure S1. XRD pattern of the simulated AB-stacking TPB-TFB COF.

Table S1. Atomistic coordinates for the refined unit cell of TPB-TFB-COF via Pawley refinement. Space group: $P1$; a = 18.8281 Å, b = 18.7227 Å, c = 3.6125 Å, $\alpha = \beta$ = 90°, and γ = 120°. Pawley-refined PXRD profile confirmed the correctness of the peak assignment as evident by the small difference in R_{wp} (4.89%) and R_p (3.69%).

C	0.1758	0.1109	1.3026
C	0.2255	0.0812	1.1543
C	0.3109	0.1285	1.1583
C	0.3507	0.2086	1.3120
C	0.3014	0.2389	1.4577
C	0.2162	0.1909	1.4558
N	0.4362	0.2614	1.3068
C	0.4874	0.2347	1.3157
H	0.1966	0.0198	1.0255
H	0.3472	0.1038	1.0317
H	0.3309	0.3008	1.5819
H	0.1800	0.2162	1.5788
C	0.9501	0.0516	1.2900
C	0.8994	0.0919	1.2857
C	0.9280	0.1712	1.4359
C	0.8798	0.2086	1.4344
C	0.8001	0.1677	1.2815
C	0.7709	0.0881	1.1338
N	0.7466	0.1998	1.2867
C	0.7723	0.2777	1.2746
H	0.9891	0.2042	1.5639
H	0.9037	0.2697	1.5621
N	0.8074	0.5719	1.2631
C	0.7296	0.5206	1.2599
C	0.8198	1.0516	1.1331
C	1.0364	1.0984	1.2973
C	1.0850	1.0605	1.2959
C	1.0453	0.9738	1.2874
C	0.9591	0.9250	1.2829
C	0.9126	0.9649	1.2846
C	0.8404	0.6579	1.2614
C	0.8006	0.6978	1.4169
C	0.8390	0.7837	1.4227
C	0.9183	0.8336	1.2755
C	0.9576	0.7933	1.1213
C	0.9201	0.7076	1.1167
H	0.7094	0.0553	1.0101
H	0.7954	0.9903	1.0079
H	1.0665	1.1660	1.2948
H	1.0823	0.9434	1.2938
H	0.8453	0.9275	1.2810
H	0.7397	0.6612	1.5428
H	0.8069	0.8127	1.5523

H	1.0190	0.8299	0.9992
H	0.9520	0.6780	0.9915
C	0.6960	0.4307	1.2732
C	0.7480	0.3968	1.2722
C	0.7160	0.3109	1.2841
C	0.6302	0.2586	1.2999
C	0.5769	0.2909	1.2996
C	0.6106	0.3771	1.2849
H	0.4669	0.1673	1.3410
H	0.6830	0.5414	1.2430
H	0.8147	0.4386	1.2606
H	0.5689	0.4023	1.2843
H	0.8393	0.3249	1.2482
H	0.6051	0.1915	1.3102

Table S2. Atomistic coordinates for the AA-stacking mode of TPB-TFB-COF optimized by using DFTB+ method. Space group: *P*1; a = 18.82811 Å, b = 18.7227 Å, c = 3.6125 Å, $\alpha = \beta$ = 90˚, and γ = 120˚.

C	0.1752	0.1094	1.2879
C	0.2226	0.0760	1.1535
C	0.3083	0.1224	1.1585
C	0.3484	0.2034	1.2976
C	0.3014	0.2372	1.4248
C	0.2157	0.1907	1.4232
N	0.4361	0.2542	1.3065
C	0.4889	0.2291	1.2740
H	0.1935	0.0148	1.0339
H	0.3424	0.0955	1.0423
H	0.3319	0.2994	1.5331
H	0.1814	0.2178	1.5371
C	0.9504	0.0510	1.2318
C	0.9012	0.0934	1.2049
C	0.9327	0.1739	1.3452
C	0.8857	0.2127	1.3392
C	0.8057	0.1718	1.1946
C	0.7740	0.0918	1.0563
N	0.7534	0.2073	1.2021
C	0.7778	0.2853	1.2382
H	0.9922	0.2057	1.4768
H	0.9108	0.2728	1.4652
N	0.8005	0.5714	1.3415
C	0.7225	0.5185	1.3628
C	0.8212	1.0532	1.0566
C	1.0365	1.0969	1.2202
C	1.0840	1.0595	1.2860
C	1.0440	0.9742	1.3500
C	0.9582	0.9257	1.3330
C	0.9120	0.9651	1.2811
C	0.8382	0.6596	1.3584
C	0.7983	0.7000	1.5021
C	0.8377	0.7863	1.5032
C	0.9174	0.8339	1.3571
C	0.9572	0.7925	1.2203
C	0.9182	0.7065	1.2260
H	0.7126	0.0601	0.9430
H	0.7954	0.9925	0.9351
H	1.0669	1.1619	1.1551
H	1.0800	0.9457	1.4180
H	0.8459	0.9288	1.2793
H	0.7379	0.6656	1.6225
H	0.8063	0.8155	1.6259
H	1.0173	0.8261	1.0943

H	0.9490	0.6757	1.1133
C	0.6924	0.4293	1.3293
C	0.7472	0.3993	1.2992
C	0.7183	0.3147	1.2621
C	0.6335	0.2595	1.2540
C	0.5779	0.2884	1.2841
C	0.6079	0.3735	1.3227
H	0.4689	0.1643	1.2464
H	0.6781	0.5389	1.3884
H	0.8127	0.4421	1.3052
H	0.5652	0.3962	1.3459
H	0.8424	0.3306	1.2496
H	0.6108	0.1940	1.2250

Table S3. Atomistic coordinates for the AB-stacking mode of TPB-TFB-COF optimized by using DFTB+ method. Space group: *P*1; a = 37.6562 Å, b = 37.4455 Å, c = 7.22 Å, $\alpha = \beta$ = 90˚, and γ = 120˚.

C	-0.0644	0.2220	0.1601
C	-0.0400	0.2103	0.0586
C	0.0028	0.2336	0.0671
C	0.0220	0.2692	0.1783
C	-0.0022	0.2811	0.2778
C	-0.0450	0.2576	0.2704
N	0.0658	0.2948	0.1867
C	0.0921	0.2825	0.1579
H	-0.0542	0.1834	-0.0299
H	0.0207	0.2245	-0.0166
H	0.0124	0.3086	0.3627
H	-0.0632	0.2669	0.3522
C	0.3235	0.1934	0.1409
C	0.2984	0.2142	0.1385
C	0.3129	0.2529	0.2237
C	0.2891	0.2720	0.2255
C	0.2503	0.2529	0.1408
C	0.2356	0.2144	0.0577
N	0.2242	0.2707	0.1415
C	0.2365	0.3097	0.1611
H	0.3423	0.2682	0.2931
H	0.3006	0.3010	0.2972
N	0.2485	0.4533	0.1492
C	0.2098	0.4272	0.1756
C	0.2594	0.1953	0.0551
C	-0.1334	0.2166	0.1416
C	-0.1098	0.1972	0.1514
C	-0.1296	0.6540	0.1552
C	0.3275	0.6301	0.1494
C	0.3044	0.6501	0.1443
C	0.2672	0.4973	0.1505
C	0.2501	0.5177	0.2467
C	0.2696	0.5607	0.2460
C	0.3068	0.5843	0.1508
C	0.3240	0.5637	0.0565
C	0.3045	0.5206	0.0577
H	0.2057	0.1995	-0.0066
H	0.2472	0.6659	-0.0146
H	-0.1180	0.2498	0.1308
H	-0.1116	0.6389	0.1691
H	0.2713	0.6318	0.1439
H	0.2221	0.5006	0.3257
H	0.2558	0.5755	0.3226
H	-0.1477	0.5808	-0.0205

H	0.3180	0.5052	-0.0159
C	0.1944	0.3824	0.1723
C	0.2216	0.3671	0.1687
C	0.2069	0.3246	0.1626
C	0.1643	0.2971	0.1589
C	0.1367	0.3120	0.1640
C	0.1520	0.3547	0.1719
H	0.0817	0.2502	0.1335
H	0.1881	0.4379	0.1931
H	0.2543	0.3883	0.1707
H	0.1309	0.3664	0.1747
H	0.2689	0.3321	0.1715
H	0.1528	0.2642	0.1535
C	0.4356	0.2220	0.1601
C	0.4600	0.2103	0.0586
C	0.5028	0.2336	0.0671
C	0.5220	0.2692	0.1783
C	0.4978	0.2811	0.2778
C	0.4550	0.2576	0.2704
N	0.5658	0.2948	0.1867
C	0.5921	0.2825	0.1579
H	0.4458	0.1834	-0.0299
H	0.5207	0.2245	-0.0166
H	0.5124	0.3086	0.3627
H	0.4368	0.2669	0.3522
C	0.8235	0.1934	0.1409
C	0.7984	0.2142	0.1385
C	0.8129	0.2529	0.2237
C	0.7891	0.2720	0.2255
C	0.7503	0.2529	0.1408
C	0.7356	0.2144	0.0577
N	0.7242	0.2707	0.1415
C	0.7365	0.3097	0.1611
H	0.8423	0.2682	0.2931
H	0.8006	0.3010	0.2972
N	0.7485	0.4533	0.1492
C	0.7098	0.4272	0.1756
C	0.7594	0.1953	0.0551
C	0.3666	0.2166	0.1416
C	0.3902	0.1972	0.1514
C	0.3704	0.6540	0.1552
C	0.8275	0.6301	0.1494
C	0.8044	0.6501	0.1443
C	0.7672	0.4973	0.1505
C	0.7501	0.5177	0.2467
C	0.7696	0.5607	0.2460
C	0.8068	0.5843	0.1508
C	0.8240	0.5637	0.0565
C	0.8045	0.5206	0.0577

H	0.7057	0.1995	-0.0066
H	0.7472	0.6659	-0.0146
H	0.3820	0.2498	0.1308
H	0.3884	0.6389	0.1691
H	0.7713	0.6318	0.1439
H	0.7221	0.5006	0.3257
H	0.7558	0.5755	0.3226
H	0.3523	0.5808	-0.0205
H	0.8180	0.5052	-0.0159
C	0.6944	0.3824	0.1723
C	0.7216	0.3671	0.1687
C	0.7069	0.3246	0.1626
C	0.6643	0.2971	0.1589
C	0.6367	0.3120	0.1640
C	0.6520	0.3547	0.1719
H	0.5817	0.2502	0.1335
H	0.6881	0.4379	0.1931
H	0.7543	0.3883	0.1707
H	0.6309	0.3664	0.1747
H	0.7689	0.3321	0.1715
H	0.6528	0.2642	0.1535
C	-0.0644	0.7220	0.1601
C	-0.0400	0.7103	0.0586
C	0.0028	0.7336	0.0671
C	0.0220	0.7692	0.1783
C	-0.0022	0.7811	0.2778
C	-0.0450	0.7576	0.2704
N	0.0658	0.7948	0.1867
C	0.0921	0.7825	0.1579
H	-0.0542	0.6834	-0.0299
H	0.0207	0.7245	-0.0166
H	0.0124	0.8086	0.3627
H	-0.0632	0.7669	0.3522
C	0.3235	0.6934	0.1409
C	0.2984	0.7142	0.1385
C	0.3129	0.7529	0.2237
C	0.2891	0.7720	0.2255
C	0.2503	0.7529	0.1408
C	0.2356	0.7144	0.0577
N	0.2242	0.7707	0.1415
C	0.2365	0.8097	0.1611
H	0.3423	0.7682	0.2931
H	0.3006	0.8010	0.2972
N	0.2485	0.9533	0.1492
C	0.2098	0.9272	0.1756
C	0.2594	0.6953	0.0551
C	-0.1334	0.7166	0.1416
C	-0.1098	0.6972	0.1514
C	-0.1296	1.1540	0.1552

C	0.3275	1.1301	0.1494
C	0.3044	1.1501	0.1443
C	0.2672	0.9973	0.1505
C	0.2501	1.0177	0.2467
C	0.2696	1.0607	0.2460
C	0.3068	1.0843	0.1508
C	0.3240	1.0637	0.0565
C	0.3045	1.0206	0.0577
H	0.2057	0.6995	-0.0066
H	0.2472	1.1659	-0.0146
H	-0.1180	0.7498	0.1308
H	-0.1116	1.1389	0.1691
H	0.2713	1.1318	0.1439
H	0.2221	1.0006	0.3257
H	0.2558	1.0755	0.3226
H	-0.1477	1.0808	-0.0205
H	0.3180	1.0052	-0.0159
C	0.1944	0.8824	0.1723
C	0.2216	0.8671	0.1687
C	0.2069	0.8246	0.1626
C	0.1643	0.7971	0.1589
C	0.1367	0.8120	0.1640
C	0.1520	0.8547	0.1719
H	0.0817	0.7502	0.1335
H	0.1881	0.9379	0.1931
H	0.2543	0.8883	0.1707
H	0.1309	0.8664	0.1747
H	0.2689	0.8321	0.1715
H	0.1528	0.7642	0.1535
C	0.4356	0.7220	0.1601
C	0.4600	0.7103	0.0586
C	0.5028	0.7336	0.0671
C	0.5220	0.7692	0.1783
C	0.4978	0.7811	0.2778
C	0.4550	0.7576	0.2704
N	0.5658	0.7948	0.1867
C	0.5921	0.7825	0.1579
H	0.4458	0.6834	-0.0299
H	0.5207	0.7245	-0.0166
H	0.5124	0.8086	0.3627
H	0.4368	0.7669	0.3522
C	0.8235	0.6934	0.1409
C	0.7984	0.7142	0.1385
C	0.8129	0.7529	0.2237
C	0.7891	0.7720	0.2255
C	0.7503	0.7529	0.1408
C	0.7356	0.7144	0.0577
N	0.7242	0.7707	0.1415
C	0.7365	0.8097	0.1611

H	0.8423	0.7682	0.2931
H	0.8006	0.8010	0.2972
N	0.7485	0.9533	0.1492
C	0.7098	0.9272	0.1756
C	0.7594	0.6953	0.0551
C	0.3666	0.7166	0.1416
C	0.3902	0.6972	0.1514
C	0.3704	1.1540	0.1552
C	0.8275	1.1301	0.1494
C	0.8044	1.1501	0.1443
C	0.7672	0.9973	0.1505
C	0.7501	1.0177	0.2467
C	0.7696	1.0607	0.2460
C	0.8068	1.0843	0.1508
C	0.8240	1.0637	0.0565
C	0.8045	1.0206	0.0577
H	0.7057	0.6995	-0.0066
H	0.7472	1.1659	-0.0146
H	0.3820	0.7498	0.1308
H	0.3884	1.1389	0.1691
H	0.7713	1.1318	0.1439
H	0.7221	1.0006	0.3257
H	0.7558	1.0755	0.3226
H	0.3523	1.0808	-0.0205
H	0.8180	1.0052	-0.0159
C	0.6944	0.8824	0.1723
C	0.7216	0.8671	0.1687
C	0.7069	0.8246	0.1626
C	0.6643	0.7971	0.1589
C	0.6367	0.8120	0.1640
C	0.6520	0.8547	0.1719
H	0.5817	0.7502	0.1335
H	0.6881	0.9379	0.1931
H	0.7543	0.8883	0.1707
H	0.6309	0.8664	0.1747
H	0.7689	0.8321	0.1715
H	0.6528	0.7642	0.1535
C	0.0799	0.0533	0.6266
C	0.1042	0.0397	0.5364
C	0.1471	0.0632	0.5434
C	0.1665	0.1008	0.6406
C	0.1424	0.1147	0.7267
C	0.0996	0.0911	0.7219
N	0.2103	0.1263	0.6487
C	0.2365	0.1134	0.6338
H	0.0900	0.0114	0.4574
H	0.1649	0.0525	0.4692
H	0.1571	0.1438	0.7997
H	0.0819	0.1022	0.7949

C	0.4672	0.0237	0.6078
C	0.4421	0.0443	0.6027
C	0.4539	0.0801	0.7079
C	0.4301	0.0992	0.7074
C	0.3940	0.0829	0.6013
C	0.3819	0.0470	0.4985
N	0.3681	0.1008	0.6028
C	0.3804	0.1394	0.6351
H	0.4810	0.0928	0.7944
H	0.4393	0.1259	0.7942
N	0.3931	0.2839	0.6336
C	0.3547	0.2577	0.6661
C	0.4059	0.0280	0.4975
C	0.0102	0.0471	0.6087
C	0.0342	0.0281	0.6215
C	0.0144	0.4849	0.6314
C	0.4714	0.4607	0.6269
C	0.4481	0.4805	0.6158
C	0.4118	0.3280	0.6364
C	0.3954	0.3484	0.7379
C	0.4148	0.3916	0.7358
C	0.4509	0.4150	0.6326
C	0.4674	0.3943	0.5336
C	0.4481	0.3513	0.5373
H	0.3539	0.0341	0.4181
H	0.3961	0.5006	0.4138
H	0.0251	0.0803	0.5959
H	0.0325	0.4699	0.6477
H	0.4149	0.4622	0.6141
H	0.3682	0.3313	0.8216
H	0.4020	0.4067	0.8172
H	-0.0052	0.4113	0.4509
H	0.4611	0.3358	0.4602
C	0.3391	0.2129	0.6546
C	0.3661	0.1973	0.6505
C	0.3511	0.1547	0.6403
C	0.3086	0.1276	0.6369
C	0.2811	0.1426	0.6398
C	0.2967	0.1854	0.6475
H	0.2259	0.0807	0.6220
H	0.3330	0.2682	0.6915
H	0.3989	0.2183	0.6556
H	0.2759	0.1974	0.6475
H	0.4127	0.1614	0.6529
H	0.2969	0.0946	0.6322
C	0.5799	0.0533	0.6266
C	0.6042	0.0397	0.5364
C	0.6471	0.0632	0.5434
C	0.6665	0.1008	0.6406

C	0.6424	0.1147	0.7267
C	0.5996	0.0911	0.7219
N	0.7103	0.1263	0.6487
C	0.7365	0.1134	0.6338
H	0.5900	0.0114	0.4574
H	0.6649	0.0525	0.4692
H	0.6571	0.1438	0.7997
H	0.5819	0.1022	0.7949
C	0.9672	0.0237	0.6078
C	0.9421	0.0443	0.6027
C	0.9539	0.0801	0.7079
C	0.9301	0.0992	0.7074
C	0.8940	0.0829	0.6013
C	0.8819	0.0470	0.4985
N	0.8681	0.1008	0.6028
C	0.8804	0.1394	0.6351
H	0.9810	0.0928	0.7944
H	0.9393	0.1259	0.7942
N	0.8931	0.2839	0.6336
C	0.8547	0.2577	0.6661
C	0.9059	0.0280	0.4975
C	0.5102	0.0471	0.6087
C	0.5342	0.0281	0.6215
C	0.5144	0.4849	0.6314
C	0.9714	0.4607	0.6269
C	0.9481	0.4805	0.6158
C	0.9118	0.3280	0.6364
C	0.8954	0.3484	0.7379
C	0.9148	0.3916	0.7358
C	0.9509	0.4150	0.6326
C	0.9674	0.3943	0.5336
C	0.9481	0.3513	0.5373
H	0.8539	0.0341	0.4181
H	0.8961	0.5006	0.4138
H	0.5251	0.0803	0.5959
H	0.5325	0.4699	0.6477
H	0.9149	0.4622	0.6141
H	0.8682	0.3313	0.8216
H	0.9020	0.4067	0.8172
H	0.4949	0.4113	0.4509
H	0.9611	0.3358	0.4602
C	0.8391	0.2129	0.6546
C	0.8661	0.1973	0.6505
C	0.8511	0.1547	0.6403
C	0.8086	0.1276	0.6369
C	0.7811	0.1426	0.6398
C	0.7967	0.1854	0.6475
H	0.7259	0.0807	0.6220
H	0.8330	0.2682	0.6915

H	0.8989	0.2183	0.6556
H	0.7759	0.1974	0.6475
H	0.9127	0.1614	0.6529
H	0.7969	0.0946	0.6322
C	0.0799	0.5533	0.6266
C	0.1042	0.5397	0.5364
C	0.1471	0.5632	0.5434
C	0.1665	0.6008	0.6406
C	0.1424	0.6147	0.7267
C	0.0996	0.5911	0.7219
N	0.2103	0.6263	0.6487
C	0.2365	0.6134	0.6338
H	0.0900	0.5114	0.4574
H	0.1649	0.5525	0.4692
H	0.1571	0.6438	0.7997
H	0.0819	0.6022	0.7949
C	0.4672	0.5237	0.6078
C	0.4421	0.5443	0.6027
C	0.4539	0.5801	0.7079
C	0.4301	0.5992	0.7074
C	0.3940	0.5829	0.6013
C	0.3819	0.5470	0.4985
N	0.3681	0.6008	0.6028
C	0.3804	0.6394	0.6351
H	0.4810	0.5928	0.7944
H	0.4393	0.6259	0.7942
N	0.3931	0.7839	0.6336
C	0.3547	0.7577	0.6661
C	0.4059	0.5280	0.4975
C	0.0102	0.5471	0.6087
C	0.0342	0.5281	0.6215
C	0.0144	0.9849	0.6314
C	0.4714	0.9607	0.6269
C	0.4481	0.9805	0.6158
C	0.4118	0.8280	0.6364
C	0.3954	0.8484	0.7379
C	0.4148	0.8916	0.7358
C	0.4509	0.9150	0.6326
C	0.4674	0.8943	0.5336
C	0.4481	0.8513	0.5373
H	0.3539	0.5341	0.4181
H	0.3961	1.0006	0.4138
H	0.0251	0.5803	0.5959
H	0.0325	0.9699	0.6477
H	0.4149	0.9622	0.6141
H	0.3682	0.8313	0.8216
H	0.4020	0.9067	0.8172
H	-0.0052	0.9113	0.4509
H	0.4611	0.8358	0.4602

C	0.3391	0.7129	0.6546
C	0.3661	0.6973	0.6505
C	0.3511	0.6547	0.6403
C	0.3086	0.6276	0.6369
C	0.2811	0.6426	0.6398
C	0.2967	0.6854	0.6475
H	0.2259	0.5807	0.6220
H	0.3330	0.7682	0.6915
H	0.3989	0.7183	0.6556
H	0.2759	0.6974	0.6475
H	0.4127	0.6614	0.6529
H	0.2969	0.5946	0.6322
C	0.5799	0.5533	0.6266
C	0.6042	0.5397	0.5364
C	0.6471	0.5632	0.5434
C	0.6665	0.6008	0.6406
C	0.6424	0.6147	0.7267
C	0.5996	0.5911	0.7219
N	0.7103	0.6263	0.6487
C	0.7365	0.6134	0.6338
H	0.5900	0.5114	0.4574
H	0.6649	0.5525	0.4692
H	0.6571	0.6438	0.7997
H	0.5819	0.6022	0.7949
C	0.9672	0.5237	0.6078
C	0.9421	0.5443	0.6027
C	0.9539	0.5801	0.7079
C	0.9301	0.5992	0.7074
C	0.8940	0.5829	0.6013
C	0.8819	0.5470	0.4985
N	0.8681	0.6008	0.6028
C	0.8804	0.6394	0.6351
H	0.9810	0.5928	0.7944
H	0.9393	0.6259	0.7942
N	0.8931	0.7839	0.6336
C	0.8547	0.7577	0.6661
C	0.9059	0.5280	0.4975
C	0.5102	0.5471	0.6087
C	0.5342	0.5281	0.6215
C	0.5144	0.9849	0.6314
C	0.9714	0.9607	0.6269
C	0.9481	0.9805	0.6158
C	0.9118	0.8280	0.6364
C	0.8954	0.8484	0.7379
C	0.9148	0.8916	0.7358
C	0.9509	0.9150	0.6326
C	0.9674	0.8943	0.5336
C	0.9481	0.8513	0.5373
H	0.8539	0.5341	0.4181

H	0.8961	1.0006	0.4138
H	0.5251	0.5803	0.5959
H	0.5325	0.9699	0.6477
H	0.9149	0.9622	0.6141
H	0.8682	0.8313	0.8216
H	0.9020	0.9067	0.8172
H	0.4949	0.9113	0.4509
H	0.9611	0.8358	0.4602
C	0.8391	0.7129	0.6546
C	0.8661	0.6973	0.6505
C	0.8511	0.6547	0.6403
C	0.8086	0.6276	0.6369
C	0.7811	0.6426	0.6398
C	0.7967	0.6854	0.6475
H	0.7259	0.5807	0.6220
H	0.8330	0.7682	0.6915
H	0.8989	0.7183	0.6556
H	0.7759	0.6974	0.6475
H	0.9127	0.6614	0.6529
H	0.7969	0.5946	0.6322

Section S3. 2D X-ray scattering patterns of TPB-TFB COF thin films

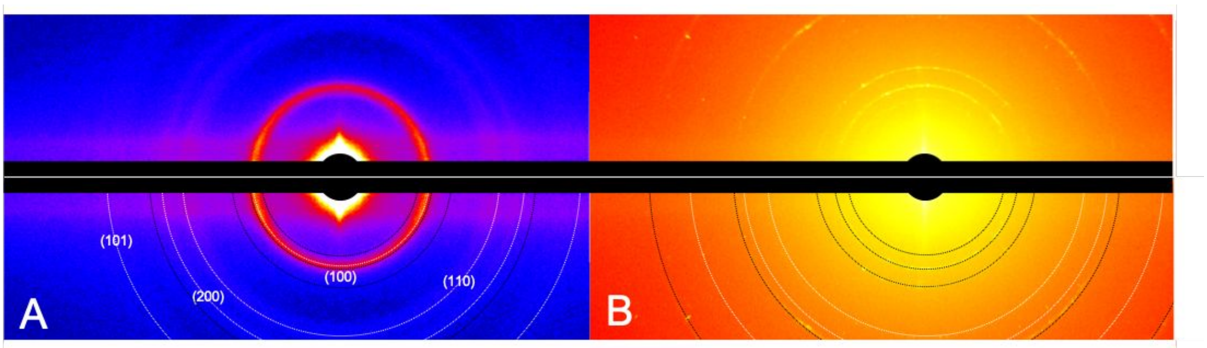

Figure S2a. 2D X-ray scattering patterns of (A) 3 μm-thick and (B) 200 nm-thick TPB-TFB COF thin films.

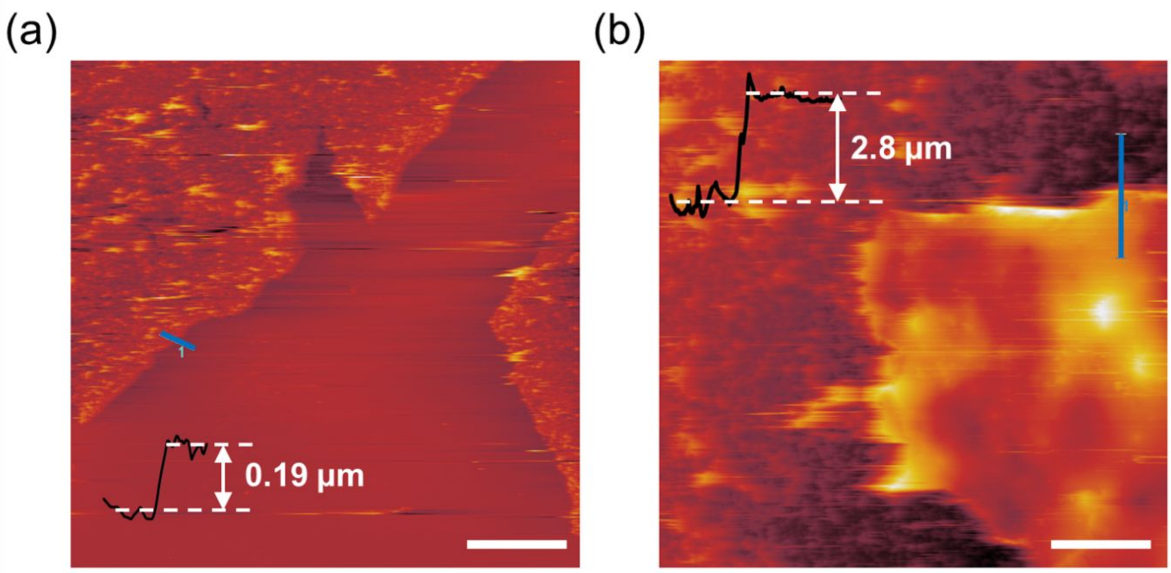

Figure S2b. AFM images of the COF films. (a) AFM image of a ~0.2 μm -thick TPB-TFB COF film. (b) AFM image of a ~3 μm-thick TPB-TFB COF film. The inserts show the height profiles and the scale bar is 20 μm.

Section S4. High-resolution transmission electron microscopic (HR-TEM) analysis of TPB-TFB COFs

Sample preparation for TEM observation of TPB-TOF COF.

Two sample preparation methods were used for TEM observation. First, TPB-TFB-COF (0.16 mg) was bath-sonicated in 8 mL of dichloromethane for 5 min, and the obtained dispersion was centrifuged at 10,000 rpm for 5 min. Then, 10 μL of the suspension was placed onto a microgrid with a lacy carbon film (NS-C15) and the excessive amount of solvent was blotted by a filter paper. This procedure was repeated 5 times. The microgrid was dried under reduced pressure for 3 hours before observation by electron microscopy. In this method, TPB-TFB-COF was not efficiently exfoliated. Nevertheless, we obtained several TEM images with periodic patterns corresponding to the structure of the COF (Fig. S3b, d-f).

We next used a temperature-swing gas exfoliation method[2] to obtain a thin film of the TPB-TFB-COF. TPB-TFB-COF (1.6 mg) was heated at 250 °C under air for 10 min, and then immediately immersed into liq. N_2. After repeating this procedure 5 times, acetonitrile (8 mL) was added and sonicated for 5 min. The obtained dispersion was centrifuged at 1,000 rpm for 10 min and 8,000 rpm for 10 min to remove unexfoliated particles. Then, 10 μL of the suspension was placed onto a microgrid with a continuous amorphous carbon film (UHR-C10) and the excessive amount of solvent was blotted by a filter paper. This procedure was repeated 5 times. The microgrid was dried under reduced pressure for 3 hours before observation by electron microscopy. In this method, we successfully observed hexagonal patterns on a thin film of TPB-TFB-COF (Fig. 1e).

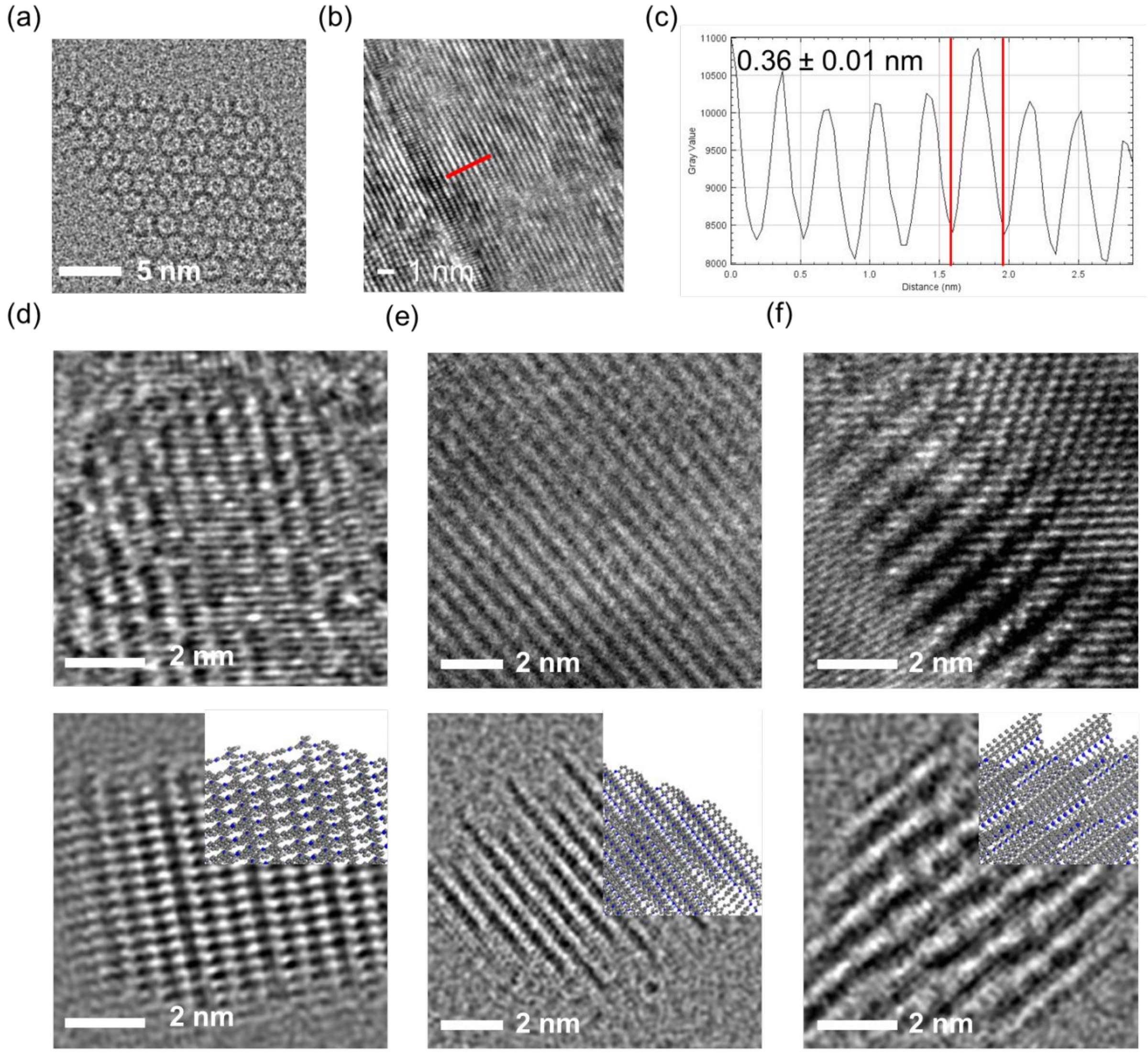

Figure S3. HR-TEM images of TPB-TFB COF. (a) Simulated TEM image of TPB-TFB-COF. (b) TEM image of a layer-stacked TPB-TFB COF. (c) The plot profile along the red line in Fig. S3b. (d-f) TEM images of TPB-TFB-COFs (Upper: TEM images, Lower: Simulated TEM images. The ZC_{LN} models[3] of TPB-TFB-COF were partially overlapped, Scale bar: 2 nm).

Section S5. Nitrogen sorption isotherms of TPB-TFB COF

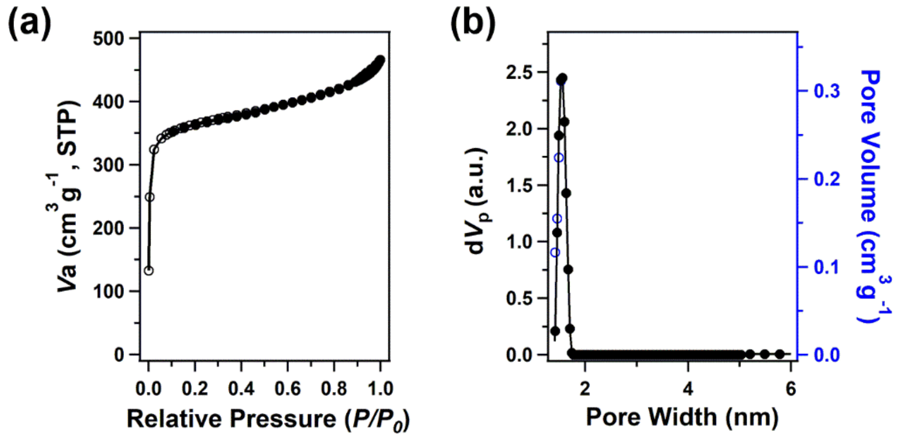

Figure S4. Nitrogen sorption isotherms of TPB-TFB COF at 77 K. (a) Absorption and desorption curves. (b) Pore size distribution and pore volume profiles based on non-local density functional theory (NLDFT) calculation.

Section S6. Optical pump-THz probe (OPTP) spectroscopy

Optical-pump THz-probe (OPTP) spectroscopy is powered by a commercial, regenerative amplified, mode-locked Ti: sapphire laser, which generates femtosecond pulsed lasers with a duration of ~50 fs, a central wavelength of 800 nm, and a repetition rate of 1 kHz. The 800 nm fundamental laser pulses are divided into three branches for optical excitation, THz generation and electro-optic sampling, respectively. The 400 nm pump pulses that used for optical excitation are produced by second harmonic generation using a β-barium borate (BBO) crystal. For THz generation, ~10% of the 800 nm fundamental laser pulses are used to produce single-cycle THz pulses with a duration of ~1 ps and a bandwidth of 1 THz by optical rectification via a 1 mm thick ZnTe (110) crystal. The generated THz pulses are focused on the samples by a pair of 90° off-axis parabolic mirrors and the time-dependent THz electrical field is detected by the electro-optic sampling method. TPB-TFB COFs in the forms of thin film (~200 nm thick, supported on the fused silica substrate) and powder (~100 μm thick, sandwiched between two fused silica substrates) are measured in the transmission geometry. Room-temperature OPTP measurements are performed under the dry N_2 environment, while low-temperature OPTP measurements are conducted under vacuum ($p < 10^{-4}$ mbar) by mounting the sample in a cryostat.

Section S7. Estimation of charge carrier lifetime

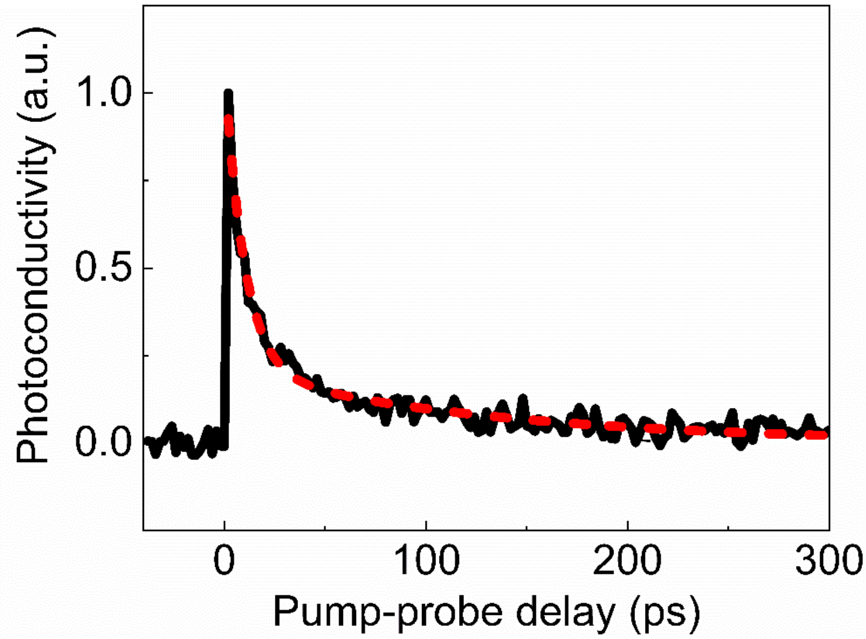

Figure S5. Time-resolved THz photoconductivity of TPB-TFB COF thin film by 400 nm photoexcitation (22 μJ·cm^{-2}).

As discussed in the main text, the relaxation process of the photoconductivity dynamics of TPB-TFB COF thin film is fitted by a bi-exponential model, as shown by equation (S1),

$$y = A_1 e^{-\frac{x}{t_1}} + A_2 e^{-\frac{x}{t_2}} \quad \text{(S1)}$$

where A_1 and t_1 represent the amplitude and time constant of the fast decay component, and A_2 and t_2 stand for the amplitude and time constant of the slow decay component. The fitting parameters are listed in **Table S4**.

Table S4. Fitting parameters of the bi-exponential model

Sample	A_1 (unitless)	t_1 (ps)	A_2 (unitless)	t_2 (ps)
TPB-TFB COF thin film	0.71913	10	0.20749	131

The average lifetimes (t) is calculated to be ~37 ps by weighting the fast and slow components following equation (S2),

$$t = \frac{A_1 t_1 + A_2 t_2}{A_1 + A_2} \quad \text{(S2)}$$

Section S8. Comparison of different TPB-TFB thin films

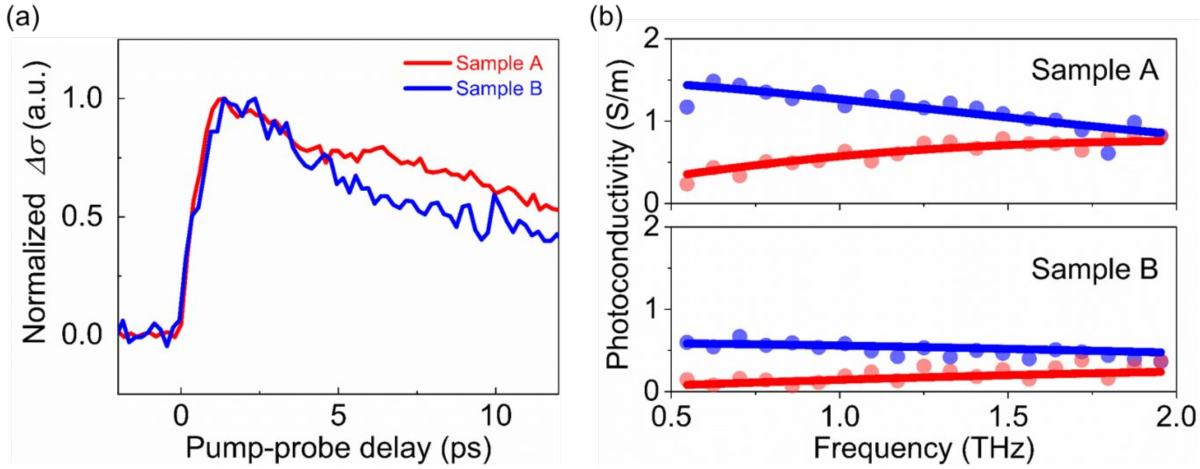

Figure S6. Comparison of different TPB-TFB thin films. (a) Time-resolved THz photoconductivity of different TPB-TFB thin films following 400 nm photoexcitation (22 μJ/cm^{-2}). (b) Frequency-resolved THz photoconductivity of different TPB-TFB COF thin films measured at 1 ps after the maximum photoconductivity at room temperature. The blue and red solid lines correspond to the Drude fits describing the real and imaginary components of the complex THz photoconductivity, respectively.

Section S9. Estimation of charge carrier diffusion length

The charge carrier diffusion length can be calculated by equation (S3),

$$L_D = \sqrt{\frac{\mu \cdot k_B \cdot T \cdot t}{e}} \tag{S3}$$

where μ, k_B, T, t, and e represent the charge mobility, Boltzmann constant, temperature, charge carrier lifetime, and elementary charge, respectively. Using the estimated charge carrier lifetime of 37 ps and charge mobility of 165 cm^2 V^{-1} s^{-1}, we calculate the charge carrier diffusion length of TPB-TFB COF thin film to be 0.13 μm at room temperature.

Section S10. Comparison of charge mobilities of 2D COFs

Table S5. Comparison of charge mobilities of 2D COFs

material	mobility ($cm^2 V^{-1} s^{-1}$)	technique
COF-366	8.1	FP-TRMC and TOF[4, a]
COF-66	3.0	FP-TRMC and TOF[4]
NiPc-BTDA COF	0.6	FP-TRMC and TOF[5]
NiPc COF	1.3	FP-TRMC and TOF[6]
2D D-A COF	0.05	FP-TRMC and TOF[7]
TPHA-PT COF	4.2	FP-TRMC and TOF[8]
H_2P-COF	3.5	FP-TRMC and TOF[9]
ZnP-COF	0.05	FP-TRMC and TOF[9]
CuP-COF	0.19	FP-TRMC and TOF[9]
HBC-COF	0.7	FP-TRMC and TOF[10]
TTF-Ph-COF	0.2	FP-TRMC and TOF[11]
TTF-Py-COF	0.08	FP-TRMC and TOF[11]
ZnPc-pz COF	4.8	Hall effect[12]
ZnPc-pz COF	2.0	THz[12]
CuPc-pz COF	0.9	Hall effect[12]
CuPc-pz COF	0.7	THz[12]
I_2-doped ZnPc-pz COF	22	Hall effect[13]
I_2-doped ZnPc-pz COF	6.3	THz[13]
HHTP-MIDA-COF	3.4	THz[14]
CuPc-MIDA-COF	13.3	THz[14]
CuPc-MIDA-COF	8.2	Hall effect[14]
DBOV-COF	0.6	THz[15]
sp^2c-COF	22.1	THz[16]
sp^2c-COF-6	2.3	THz[16]
sp^2c-COF-8	<0.1	THz[16]
sp^2c-COF-9	5.8	THz[16]
I_2-doped sp^2c-COF	51.1	THz[16]
I_2-doped sp^2c-COF-6	5.7	THz[16]
**TPB-TFB COF (this work)**	**165**	**THz**

[a]FP-TRMC and TOF, flash-photolysis time-resolved microwave and time-of-flight transient at different bias voltages.

Section S11. Comparison of charge transport properties of molecular and polymeric materials characterized by THz spectroscopy

Table S6. Comparison of charge transport properties of molecular and polymeric materials characterized by THz spectroscopy

material	mobility (cm^2 V^{-1} s^{-1})	scattering time (fs)	carrier lifetime (ps)	diffusion length (nm)
ZnPc-pz COF[12]	2.0	30	~2	~3
CuPc-pz COF[12]	0.7	30	~2	~2
I_2-doped ZnPc-pz COF[13]	6.3	66	~5	~9
HHTP-MIDA-COF[14]	3.4	16	~2	~4
CuPc-MIDA-COF[14]	13.3	23	~2	~8
DBOV-COF[15]	0.6	36	8	~4
sp^2c-COF[16]	22.1	41	~2	~11
sp^2c-COF-6[16]	2.3	30	~2	~3
sp^2c-COF-8[16]	<0.1	<5	~2	<1
sp^2c-COF-9[16]	5.8	43	~2	~5
I_2-doped sp^2c-COF[16]	51.1	83	~2	~16
I_2-doped sp^2c-COF-6[16]	5.7	67	~2	~5
c-HBC-COF[17]	44	87	~2	~15
PA-doped PANI[18]	1	<10	~5	~4
C4-DPP-BP[19]	~0.7	6-9	~2	~2
6-CZGNR-(2,1)[20]	18	29	~2	~10
HBC-6Ph[21]	31	36	~2	~13
**TPB-TFB COF (this work)**	**165**	**72**	**~40**	**~130**

References

(1) Hosokawa, F.; Shinkawa, T.; Arai, Y.; Sannomiya, T. Benchmark Test of Accelerated Multi-Slice Simulation by GPGPU. *Ultramicroscopy* **2015**, *158*, 56–64.

(2) Dong, J.; Li, X.; Peh, S. B.; Yuan, Y. Di; Wang, Y.; Ji, D.; Peng, S.; Liu, G.; Ying, S.; Yuan, D.; Jiang, J.; Ramakrishna, S.; Zhao, D. Restriction of Molecular Rotors in Ultrathin Two-Dimensional Covalent Organic Framework Nanosheets for Sensing Signal Amplification. *Chem. Mater.* **2019**, *31*, 146–160.

(3) Xing, J.; Takeuchi, K.; Kamei, K.; Nakamuro, T.; Harano, K.; Nakamura, E. Atomic-Number (Z)-Correlated Atomic Sizes for Deciphering Electron Microscopic Molecular Images. *Proc. Natl. Acad. Sci.* **2022**, *119*, e2114432119.

(4) Wan, S.; Gándara, F.; Asano, A.; Furukawa, H.; Saeki, A.; Dey, S. K.; Liao, L.; Ambrogio, M. W.; Botros, Y. Y.; Duan, X.; Seki, S.; Stoddart, J. F.; Yaghi, O. M. Covalent Organic Frameworks with High Charge Carrier Mobility. *Chem. Mater.* **2011**, *23*, 4094–4097.

(5) Ding, X.; Chen, L.; Honsho, Y.; Feng, X.; Saengsawang, O.; Guo, J.; Saeki, A.; Seki, S.; Irle, S.; Nagase, S.; Parasuk, V.; Jiang, D. An N-Channel Two-Dimensional Covalent Organic Framework. *J. Am. Chem. Soc.* **2011**, *133*, 14510–14513.

(6) Ding, X.; Guo, J.; Feng, X.; Honsho, Y.; Guo, J.; Seki, S.; Maitarad, P.; Saeki, A.; Nagase, S.; Jiang, D. Synthesis of Metallophthalocyanine Covalent Organic Frameworks That Exhibit High Carrier Mobility and Photoconductivity. *Angew. Chem. Int. Ed.* **2011**, *50*, 1289–1293.

(7) Feng, X.; Chen, L.; Honsho, Y.; Saengsawang, O.; Liu, L.; Wang, L.; Saeki, A.; Irle, S.; Seki, S.; Dong, Y.; Jiang, D. An Ambipolar Conducting Covalent Organic Framework with Self-sorted and Periodic Electron Donor-acceptor Ordering. *Adv. Mater.* **2012**, *24*, 3026–3031.

(8) Guo, J.; Xu, Y.; Jin, S.; Chen, L.; Kaji, T.; Honsho, Y.; Addicoat, M. A.; Kim, J.; Saeki, A.; Ihee, H.; Seki, S.; Irle, S.; Hiramoto, M.; Gao, J.; Jiang, D. Conjugated Organic Framework with Three-Dimensionally Ordered Stable Structure and Delocalized π Clouds. *Nat. Commun.* **2013**, *4*, 2736.

(9) Feng, X.; Liu, L.; Honsho, Y.; Saeki, A.; Seki, S.; Irle, S.; Dong, Y.; Nagai, A.; Jiang, D. High-rate Charge-carrier Transport in Porphyrin Covalent Organic Frameworks: Switching from Hole to Electron to Ambipolar Conduction. *Angew. Chem. Int. Ed.* **2012**, *51*, 2618–2622.

(10) Dalapati, S.; Addicoat, M.; Jin, S.; Sakurai, T.; Gao, J.; Xu, H.; Irle, S.; Seki, S.; Jiang,

D. Rational Design of Crystalline Supermicroporous Covalent Organic Frameworks with Triangular Topologies. *Nat. Commun.* **2015**, *6*, 7786.

(11) Jin, S.; Sakurai, T.; Kowalczyk, T.; Dalapati, S.; Xu, F.; Wei, H.; Chen, X.; Gao, J.; Seki, S.; Irle, S.; Jiang, D. Two-dimensional Tetrathiafulvalene Covalent Organic Frameworks: Towards Latticed Conductive Organic Salts. *Chem. Eur. J.* **2014**, *20*, 14608–14613.

(12) Wang, M.; Ballabio, M.; Wang, M.; Lin, H.-H.; P. Biswal, B.; Han, X.; Paasch, S.; Brunner, E.; Liu, P.; Chen, M.; Bonn, M.; Heine, T.; Zhou, S.; Cánovas, E.; Dong, R.; Feng, X. Unveiling Electronic Properties in Metal–Phthalocyanine-Based Pyrazine-Linked Conjugated Two-Dimensional Covalent Organic Frameworks. *J. Am. Chem. Soc.* **2019**, *141*, 16810–16816.

(13) Wang, M.; Wang, M.; Lin, H.-H.; Ballabio, M.; Zhong, H.; Bonn, M.; Zhou, S.; Heine, T.; Cánovas, E.; Dong, R.; Feng, X. High-Mobility Semiconducting Two-Dimensional Conjugated Covalent Organic Frameworks with p-Type Doping. *J. Am. Chem. Soc.* **2020**, *142*, 21622–21627.

(14) Jin, E.; Geng, K.; Fu, S.; Yang, S.; Kanlayakan, N.; Addicoat, M. A.; Kungwan, N.; Geurs, J.; Xu, H.; Bonn, M.; Wang, H. I.; Smet, J.; Kowalczyk, T.; Jiang, D. Exceptional Electron Conduction in Two-Dimensional Covalent Organic Frameworks. *Chem* **2021**, *7*, 3309.

(15) Jin, E.; Fu, S.; Hanayama, H.; Addicoat, M. A.; Wei, W.; Chen, Q.; Graf, R.; Landfester, K.; Bonn, M.; Zhang, K. A. I.; Wang, H. I.; Müllen, K.; Narita, A. A Nanographene-Based Two-Dimensional Covalent Organic Framework as a Stable and Efficient Photocatalyst. *Angew. Chem. Int. Ed.* **2021**, e202114059.

(16) Jin, E.; Geng, K.; Fu, S.; Addicoat, M. A.; Zheng, W.; Xie, S.; Hu, J.-S.; Hou, X.; Wu, X.; Jiang, Q.; Xu, Q.-H.; Wang, H. I.; Jiang, D. Module-Patterned Polymerization towards Crystalline 2D Sp2-Carbon Covalent Organic Framework Semiconductors. *Angew. Chem. Int. Ed.* **2021**, e202115020.

(17) Xing, G.; Zheng, W.; Gao, L.; Zhang, T.; Wu, X.; Fu, S.; Song, X.; Zhao, Z.; Osella, S.; Martínez-Abadía, M.; Wang, H. I.; Cai, J.; Mateo-Alonso, A.; Chen, L. Nonplanar Rhombus and Kagome 2D Covalent Organic Frameworks from Distorted Aromatics for Electrical Conduction. *J. Am. Chem. Soc.* **2022**, *144*, 5042–5050.

(18) Ballabio, M.; Zhang, T.; Chen, C.; Zhang, P.; Liao, Z.; Hambsch, M.; Mannsfeld, S. C. B.; Zschech, E.; Sirringhaus, H.; Feng, X.; Bonn, M.; Dong, R.; Cánovas, E. Band-like Charge Transport in Phytic Acid-Doped Polyaniline Thin Films. *Adv. Funct. Mater.*

2021, 2105184.

(19) Ohta, K.; Tokonami, S.; Takahashi, K.; Tamura, Y.; Yamada, H.; Tominaga, K. Probing Charge Carrier Dynamics in Porphyrin-Based Organic Semiconductor Thin Films by Time-Resolved THz Spectroscopy. *J. Phys. Chem. B* **2017**, *121*, 10157–10165.

(20) Wang, X.; Ma, J.; Zheng, W.; Osella, S.; Arisnabarreta, N.; Droste, J.; Serra, G.; Ivasenko, O.; Lucotti, A.; Beljonne, D.; Bonn, M.; Liu, X.; Hansen, M. R.; Tommasini, M.; De Feyter, S.; Liu, J.; Wang, H. I.; Feng, X. Cove-Edged Graphene Nanoribbons with Incorporation of Periodic Zigzag-Edge Segments. *J. Am. Chem. Soc.* **2022**, *144*, 228–235.

(21) Zeng, C.; Zheng, W.; Xu, H.; Osella, S.; Ma, W.; Wang, H. I.; Qiu, Z.; Otake, K.; Ren, W.; Cheng, H.; Müllen, K.; Bonn, M.; Gu, C.; Ma, Y. Electrochemical Deposition of a Single-Crystalline Nanorod Polycyclic Aromatic Hydrocarbon Film with Efficient Charge and Exciton Transport. *Angew. Chem. Int. Ed.* **2021**, e202115389.
